# An Enactive–Ecological Model to Guide Patient-Centered Osteopathic Care

**DOI:** 10.3390/healthcare10061092

**Published:** 2022-06-12

**Authors:** Francesco Cerritelli, Jorge E. Esteves

**Affiliations:** 1 Clinical-Based Human Research Department, Foundation COME Collaboration, 66100 Pescara, Italy; osteojorge@gmail.com; 2 Malta ICOM Educational, GZR 1071 Gzira, Malta

**Keywords:** touch, pain, free energy principle, patient-practitioner dyad, active inference, ecological niche, enactivism

## Abstract

Osteopaths commonly face complexity and clinical uncertainty in their daily professional practice as primary contact practitioners. In order to effectively deal with complex clinical presentations, osteopaths need to possess well-developed clinical reasoning to understand the individual patient’s lived experience of pain and other symptoms and how their problem impacts their personhood and ability to engage with their world. We have recently proposed (En)active inference as an integrative framework for osteopathic care. The enactivist and active inference frameworks underpin our integrative hypothesis. Here, we present a clinically based interpretation of our integrative hypothesis by considering the ecological niche in which osteopathic care occurs. Active inference enables patients and practitioners to disambiguate each other’s mental states. The patients’ mental states are unobservable and must be inferred based on perceptual cues such as posture, body language, gaze direction and response to touch and hands-on care. A robust therapeutic alliance centred on cooperative communication and shared narratives and the appropriate and effective use of touch and hands-on care enable patients to contextualize their lived experiences. Touch and hands-on care enhance the therapeutic alliance, mental state alignment, and biobehavioural synchrony between patient and practitioner. Therefore, the osteopath–patient dyad provides mental state alignment and opportunities for ecological niche construction. Arguably, this can produce therapeutic experiences which reduce the prominence given to high-level prediction errors—and consequently, the top-down attentional focus on bottom-up sensory prediction errors, thus minimizing free energy. This commentary paper primarily aims to enable osteopaths to critically consider the value of this proposed framework in appreciating the complexities of delivering person-centred care.

## 1. Introduction

Current evidence from musculoskeletal care, neuroscience, pain and cognitive science, and philosophy provides opportunities for developing and implementing robust models of osteopathic person-centred care [1]. In recent years, enactivism, predictive processing, and the Free Energy Principle (FEP) have emerged as promising frameworks for the understanding of cognition, pain, and mental health (e.g., [2,3,4,5,6,7]). We have recently argued that the osteopathic-underpinning conceptual framework aligns with an enactive and ecological take on life and mind [8]. Specifically, we have argued that the concepts of unity of body and mind, its adaptive and self-regulatory mechanisms and the person’s dynamical engagement with their world as agents can be interpreted and researched under the theoretical frameworks of enactivism, predictive processing, the FEP, and active inference [8,9,10]. Indeed, Bruineberg and colleagues [10] have argued that the FEP and the enactivist and ecological approaches to life and mind provide a robust framework for fully understanding the whole organism–environment system and their dynamical interactions. Moreover, Cormack and co-workers (2022) have recently proposed that an enactive–biopsychosocial approach to patient care enabled practitioners to appreciate the complexities and wholeness of human experience that are intrinsically embedded and embodied in an environment [11]. We argue that the enactive approach to cognition, predictive processing, FEP, and active inference provides the tools for developing a robust osteopathic person-centred care model [8,12,13]. This commentary paper builds upon our ongoing research and recently published work to provide a synopsis of critical concepts from the literature on enactivism, predictive processing, FEP, and active inference and a framework that can inform clinical reasoning in osteopathy. Despite the growing support for the use of these theoretical frameworks in clinical practice [6,7,8,11] we are acutely aware of the debate around the putative incompatibilities between enactivism and the FEP (e.g., DiPaolo et al., 2022 [14]) and the failure of predictive processing as a unified theory of cognition [15]. Although we agree that one should avoid applying these frameworks to clinical practice in an uncritical manner, there is growing empirical evidence that, for example, predictive processing and active inference have neurobiological correlations that are relevant to practitioners (e.g., Horing and Buchel, 2022 [16]). Active inference is also aligned with enactivist theories, which emphasize the self-organization of behaviour and autopoietic interactions with the environment required to enable living organisms to stay within appropriate bounds [17]. Moreover, an enactive–ecological approach addresses the limitations and misapplications of the biopsychosocial model, thus providing a “big picture” framework which enables practitioners to better understand their patients’ complex and changing experiences [11]. We start with a brief introduction to enactivism, predictive processing and active inference to provide a robust framework for our take-home messages to osteopaths. This commentary paper primarily aims to enable osteopaths to critically consider the value of this proposed framework in appreciating the complexities of delivering person-centred care. We are aware that our putative framework is mainly theoretical, thus requiring significant empirical work to develop further and validate an enactive–ecological model of osteopathic care.

Enactivism is a position in cognitive science that departs from the traditional representationalist views to consider that cognition arises from the dynamic interactions between an embodied organism and its world [17]. Instead of focusing on the mind, the body or a particular system, the enactive approach postulates that the whole individual and their dynamic interactions in their environment enact their lived experiences. Through this lens, cognition is an embodied and embedded activity that is extended in the environment [17]. Therefore, the relationship between physiological and lived experiences includes the person’s relationships with their environments [2]. Consequently, enactivism departs from the mind–body relationship to stress the mereological co-dependence between the mind, body, and world, i.e., the physiological, experiential, and environmental/sociocultural dimensions are all part of one person–world system [2]. Autopoiesis, autonomy, and sense-making are the three central concepts defining enactivism [18]. Crucially, all living includes some form of cognition or sense-making. 

Sense-making, an organism’s evaluative interaction with its environment, is one of its critical concepts [2]. Sense-making activity discloses the world as a field of relevant affordances [2]. Affordances are opportunities for action provided to the organism by its environment, e.g., hitting a ball with a bat, opening a door, or lifting a box [19,20]. As such, altered sense-making discloses an altered field of relevant affordances [2]. We argue that sense-making is particularly relevant to osteopathy and health care. A recent interpretation of pain supports our argument. Pain is conceptualized as a sense-making process that occurs through a lived body that is dynamically coupled with the world we interact with and shape [6]. 

Interestingly, in his early work, J. M. Littlejohn proposed an osteopathic model of care grounded on four fundamental tenets: adaptation, function, environment, and immunity [21]. In particular, he emphasized the body’s functional adaptation to its surrounding environment [22]. Although some of these early concepts were lost in favour of causality-based models of osteopathic care, we would argue that enactivism, FEP, predictive processing, and active inference provide opportunities to reinterpret Littlejohn’s early ideas in a critical and evidence-informed way.

Predictive coding stems from the pioneering work of Hermann von Helmholtz [23] and is predicated on the assumption that a complex system such as the brain has a generative or internal model for inferring the causes of incoming sensations. From this perspective, the brain is a Bayesian device constantly making predictions about the causes of sensory evidence, and these predictions cascade in a top-down fashion through the brain’s perceptual hierarchy [20,24]. The bottom-up sensory signals which are conveyed to the brain tend to maintain these top-down predictions linked to their causes [20,24]. The incoming sensory signals function as ‘prediction errors’, registering the difference between what the brain’s predictions are and what it receives at every level of processing. By modulating top-down predictions and consequently suppressing bottom-up prediction errors, the brain’s perceptual inferences maintain their grip on their causes in the world. Through this lens, perception is viewed as an ongoing process of prediction error minimisation [20]. Over the last few years, predictive coding has been incorporated into predictive processing, which has become a significant theory in cognitive science, neuroscience, and philosophy [25]. Predictive processing extends the scope of predictive coding to include the consequences of action. Therefore, Bayesian belief updating is not strictly based on perceptual inference but also considers active inference’s crucial role.

Predictive processing views the brain as a hierarchical, multilevel prediction machine, and therefore what we feel, see, and hear is the brain’s best guess of the causes of its sensations [20,26]. Crucially, the embodied brain is a generative model of the body in the world that is utilized to generate predictions of the sensory outcomes of the organism’s actions in the world. Therefore, the embodied brain anticipates the distal causes of interoceptive, exteroceptive, and proprioceptive signals to minimize prediction errors [26,27]. These causes may involve, for example, motor or autonomic action or interoceptive sensations. Perception is a proactive process; the brain does not passively react to the world. Instead, it develops hypotheses and proactively tests them against sensory evidence [9]. Prediction error minimization occurs by either perceptual or active inference. Perceptual inference requires updating the generative model in response to prediction errors. In contrast, active inference requires the agent to act on their world to generate predicted or anticipated sensations [28].

Sense-making, autopoiesis, and adaptivity have been considered key concepts for enactivists for interpreting how biological systems access their environments. Although we endorse this position, it is also crucial to understand the life–mind continuity under the FEP [29]. The FEP provides a formal analysis for autopoiesis and active inference properties. Yet the FEP is enacted by all systems with a Markov blanket [29]. Given stable boundary conditions, dynamical systems spontaneously self-organize to maximize evidence for their enacted world model by minimizing their expected variational free energy [30]. To minimize free energy or prediction errors, organisms must possess a generative model for inferring how exteroceptive, interoceptive, and proprioceptive sensations are generated [3,20]. This generative model enables biological systems to narrow the gap between predicted and incoming sensory signals by altering their sensory states via action or altering predictions via perception [3,20,30].

Predictive coding and active inference can all be understood through the lens of FEP as stemming from what it means to exist and be alive [20]. Autonomy is necessary for describing living systems’ behaviours under the umbrella of Bayesian inference and free energy minimization [5,31]. Although the FEP endorses the hypothesis of the Bayesian brain, anticipating and changing the consequences of actions and reducing bound entropy production requires acting on the world; this differentiates the FEP from the predictive coding framework [32]. Active inference illustrates the fundamental tendency of biological systems to comply by generating, revising, and preserving inferences about the environment whilst maintaining a distinction between themselves and their environment [5]. The biological system is simply an actively maintained boundary—a Markov blanket—that through active inference differentiates the internal states from the external environment [30,33,34]. Because Markov blankets ease the internal–external state exchange, they imply action–perception cycles as the biological system engages with the environment based on the available affordances it has been given [34]. The concept of Markov blankets has been used to illustrate the critical precondition for any adaptive system to have some separation and autonomy from the environment [32]. However, Bruineberg and colleagues (2022) have recently pointed out some inaccuracies in the literature regarding the use of the term [35]. The authors propose a distinction between ‘Pearl Blankets’—the original epistemic use of Markov blankets as a tool for Bayesian inference, and ‘Friston Blankets’ to refer to the metaphysical construct in the FEP framework to demarcate the physical boundary between an agent and its environment [35].Although this is an important debate in the literature, it is beyond the purpose of this paper, and we will therefore maintain the use of Markov blankets to illustrate our arguments.

Crucially, active inference goes beyond the concept of the embodied brain, reducing the uncertainty surrounding its sensory evidence via perceptual inference to consider the role of an embodied agent actively and selectively sampling the world or generative model [36]. To this end, active inference puts the action into perception considering planning as inference, i.e., inferring what one would do next to resolve uncertainty regarding their lived world [37]. The human being is a complex, dynamic, open, system, and any change to one component of one’s personhood can have an impact on their physical and mental health and well-being. Thus, a critical understanding of the FEP and active inference is fundamental to our clinical reasoning framework in osteopathy. The organism, its body, the embodied brain, and the world constitute the generative model and will determine the probabilities of the kind of actions one can engage in [31]. This can be interpreted through the conceptual notion of an Umwelt, in which an organism’s world is itself a constituting and constraining feature of its embodiment [31]. Active inference provides a formal theoretical framework for understanding the constitutive coupling of the brain–body–environment [10,31,38]. Within this context, it is possible to argue that the generative model might be regarded as a model that comprises the entire embodied organism, where the latter considers all the living elements within and outside the human being [38]. Therefore, as a consequence, active inference might be considered as shaping the organism’s (or agent’s) conscious and lived experience [38]. This perspective is consistent with embodied–enactive approaches and enables the dialectic between what an organism is (embodiment) and what it does (enactment). As a result, it can be argued that active inference is enactive inference [39]. (En)active inference portrays how complex dynamical systems develop adaptive agency independently [40]. Crucially, people do not simply try to make sense of their sensations; they actively create their own sensoria [37]. Therefore, (En)active inference provides a framework for comprehending health and illness and, potentially, providing person-centred osteopathic care.

## 2. A Critical Evaluation of Osteopathic Models and How They Fit into This Putative Framework

Enactivism, predictive processing, and active inference have implications for clinical practice. In support of this thesis, Owens and colleagues proposed that bottom-up interoceptive prediction errors may be a source of anxiety and may drive responses in the autonomic, metacognitive, motor homeostatic, and allostatic systems [41]. Similarly, Barrett and colleagues [42] argued that depression is the outcome of a brain insensitive to prediction errors coupled with inefficient energy regulation associated with intense suffering and difficulties in participating in vigorous mental or physical activity. Treatments aimed at re-categorizing prior beliefs provide a vital therapeutic intervention. On this point, cognitive behavioural therapy enables individuals to develop new concepts that as predictive signals alter the gain in prediction errors via the salience network. Over time, this process may modify inputs, ultimately becoming the ‘empirical priors’ that agranular limbic cortices use to initiate subsequent predictions [42]. From an enactivist perspective, De Haan proposed that mental health conditions such as depression should be viewed as disorders of sense-making [43]. From this perspective, the therapeutic encounter enables the recognition of rigid and inappropriate patterns of interaction and the practice of alternative strategies for engaging with the world [8,43].

**Figure 1:**
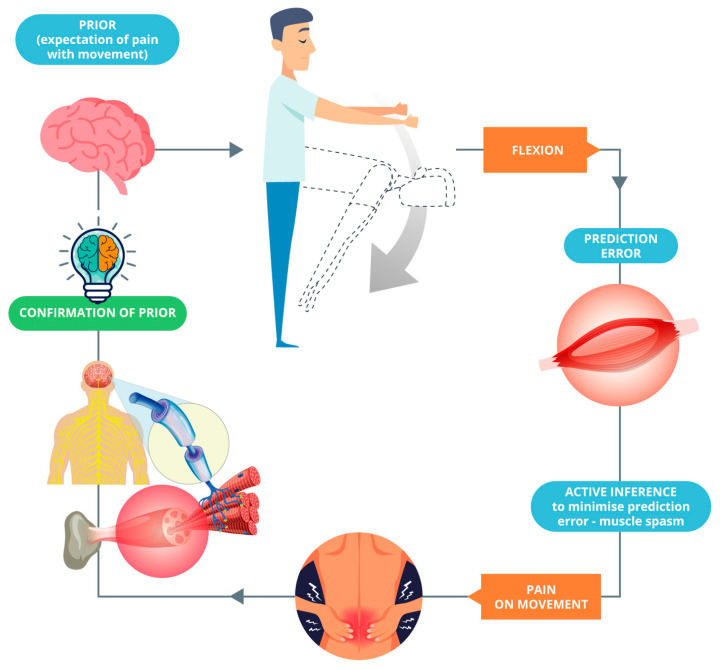
The expectation of pain (prior) is confirmed by the movement (confirmation of prior) through the active inference.

**Figure 2:**
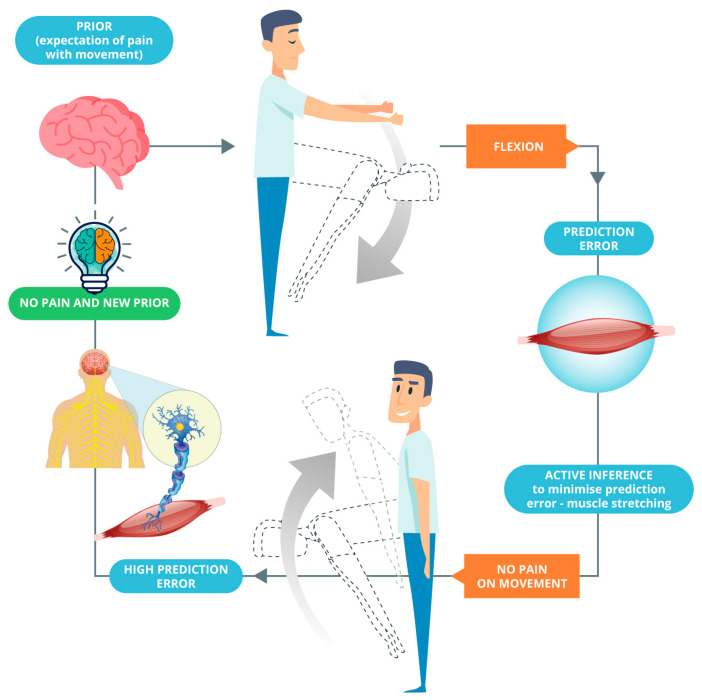
The expectation of pain (prior) is violated by feeling no pain during the movement, generating high prediction error and a new prior. This is argued to be the cycle established after a manual osteopathic treatment.

Apart from depression and anxiety, predictive processing and active inference are very relevant to patients with, for example, persistent pain and kinesiophobia; protective mechanisms may be associated with movements initiated to reduce prediction errors and confirm existing predictions (Figure 1). To this end, the purpose of an intervention would be to cause positive surprise and a high prediction error that will violate existing predictions and update the brain’s internal model (see Bohlen et al. [12], on this point). This can be achieved using hands-on care, cognitive reassurance, appropriate language, and graded exposure to movement and interoceptive sensations. We argue that these strategies can update the brain’s internal model whilst reducing activity in regions including the amygdala (critical concerning signal uncertainty) and achieving allostatic regulation (Figure 2). On this point, we have recently proposed that from an active inference standpoint, the essence of touch in osteopathy is the provision of sensory data to be (re)interpreted by a patient [44]. Practitioners should weaken their patients’ pre-existing generative models through flattening sensory states, changing the sense of agency, and facilitating the redeployment of attention. Therefore, a new robust generative model can be installed through inner communication complemented with hands-on care as the sensory evidence for this new narrative [44].

Here, we propose a model for clinical reasoning in osteopathy that fuses the profession’s conceptual basis with the enactivist, predictive processing, FEP, and active inference frameworks and evidence from the fields of interoception, allostatic regulation, and affective touch. Arguably, in osteopathy, the concepts of body-mind unity, adaptation, and self-regulation are aligned with the principles of sense-making and autopoiesis central to enactivism [1]. Osteopathy should move further and over the concept of a therapy centred to the body, which is informed by, arguably, models of care, including the biomedical. Indeed, human behaviour and function are complex, individual, and difficult to predict. Therefore, osteopaths should evaluate their patients within an inconstant ecological system [45]. Ultimately, health and disease should be interpreted within the person’s environment, which includes life and the way in which the person interacts with the external world [45,46].

The model enables practitioners to effectively evaluate their patients within a biopsychosocial(existential) framework whilst considering how their embodied brains construct their internal models of disease, health, and well-being. The model also enables practitioners to implement treatment strategies underpinned by effective language and affective touch and integrated with cognitive reassurance; graded exposure to movement, exercise, and interoceptive sensations; and mindfulness-based strategies. From a clinical standpoint, chronic pain treatment is an excellent example of the ecological–enactive and active inference paradigms: underpinning embodied action is pain, which reflects the body’s and the world’s uncertainty [47,48]. This ultimately alters interactions between organisms or agents and their environments either temporarily, such as in acute pain, or permanently in cases of chronic pain [49]. In subjects with chronic pain, other physical symptomatology, and associated kinesiophobia, an osteopathic intervention would cause a positive surprise and a high precision-weighted prediction error that will violate existing predictions (for example, fear of movement in lumbar flexion) (Figure 2). This can be achieved through incoming interoceptive, exteroceptive, or proprioceptive sensory sensations or by reframing existing beliefs and expectations based on new information. Arguably, through repeated steps of perceptual inference across the hierarchy, the therapeutic intervention updates the brain’s internal generative model. This process is illustrated in Equation (1). It is essential to highlight that the purpose of Equations (1) and (2) adapted from Hohwy (2020) [25] is simply to illustrate the process of perceptual inference rather than for empirical purposes.

New prediction ^(“I can bend forwards with less pain”)^ = Old prediction ^(“lumbar flexion increases my pain”)^ + Osteopathic treatment ^(learning rate × Prediction error from interoceptive, exteroceptive and proprioceptive inputs)^ (1)

On occasion, patients may refer to sensations of ‘good pain’. In this context, high-precision weighted predictions will indicate that the source of the noxious stimulation is the osteopath; forecasts of pain can, therefore, be achieved without engaging low-level motor reflexive responses. This will likely lead to allostatic changes in the form of updated beliefs about the “safety” and tolerance of nociceptive signals arising from applying an osteopathic technique (see Von Mohr and Fotopoulou [50], on this point). This process is illustrated in Equation (2).

New prediction ^(“The treatment was uncomfortable, but I feel much better”)^ = Old prediction ^(“movement and touch are painful”)^ + Osteopathic treatment delivered by a trusted practitioner − “I’m in safe hands” ^(learning rate × Prediction error from interoceptive, exteroceptive and proprioceptive inputs and sense of safety)^ (2)

We propose that osteopathic care be viewed as an interactive ritual that enables sensations to be reinterpreted, attention redirected, and distracting stimuli attenuated and ignored. Therefore, osteopaths must recognise patterns of interaction in their patients that show inflexibility and inappropriateness; this will enable osteopaths to implement therapeutic strategies that can help individuals interact with the environment, considering their patients’ symptoms and personalities [50]. Therefore, the practice of osteopathy might be conceived as participatory sense-making [43]. 

## 3. A Clinical Case Example

Consider the following scenario: a 48-year-old female with chronic neck, upper thoracic back, and epigastric pain; headaches; and sporadic abdominal discomfort. Aside from that, she has a history of gastro-oesophageal reflux disease, anxiety-related problems, and a fibromatous uterus that was discovered accidentally. After seeing a variety of health care professionals, including a cardiologist, gynaecologist, gastroenterologist, neurologists, physiotherapists, and osteopaths, she decided to discontinue treatment due to the slight improvement in her symptoms. Although she has received assurances from her doctors that everything is fine, she is concerned that she may have breast cancer or a heart condition. She has been told that degenerative changes in her cervical spine cause her neck pain and that there is nothing wrong with her heart or breasts. Following the clinical evaluation, the osteopath arrives at the conclusion that the patient suffers from chronic non-specific neck pain with likely central and peripheral nociplastic changes and maladaptive cognitions. On examination, the clinician finds areas of protective muscle spasm in the neck and upper back regions with associated allodynia and signs of fear of movement in her cervical spine. In their examination, the clinician also finds evidence of a temporomandibular disorder and generalised sensitivity to the palpation of her abdomen. Particularly relevant to this scenario is the concept of cognitive immunisation [51]. Patients with persistent physical symptoms like the one portrayed here are likely to develop dysfunctional expectations about health and disease and become progressively immune to reassurance through cognitive reappraisal [52]. From a predictive processing perspective, the process of cognitive immunisation against disconfirmatory evidence and reassurance corresponds to too much precision afforded to prior predictions [52]. Strong priors override benign bodily signals, making a person believe something serious is wrong with their body [52]. Therefore, we argue that the purpose of a therapeutic intervention is to cause a positive surprise and a high prediction error that will violate existing predictions and update the brain’s generative model.

This scenario exemplifies the complexity that many osteopaths and other health care practitioners must deal with in their daily clinical practice. The patient claims that she is constantly conscious of her neck and chest pain. When we are in good health, the interaction with the world generates sensorial pleasures through our embodied organisms, and we do not have to think about them very much when we are sick [53]. As part of filtering and making sense of sensory input, the body is used by our brain to collect information and give a meaning to it, making a contribution to our personhood and identity. As a result, one could argue that brains are embodied, communicating with their environment via an ‘invisible’ body. Even when one’s body appears to be in good health and well-being, the body typically ‘reappears’ when one is in pain or suffering from some form of dysfunction [54]. In this way, pain impacts the foundation that underpins one’s selfhood [53]. When presenting a case of long-lasting pain and other physical symptomatology, it is critical to note that the body, in addition to becoming ‘visible’, evolves into the focus of attention [55]. When an individual pays only partial attention to their body, it impairs their ability to engage in environmental interaction, including with other people, in other words, their sense of agency. Diseases develop into an agency loss, and the individual’s inability to carry out goal-oriented behaviour in a predictable manner represents the start of their transformation into a patient (or patientization) [45].

Considering the above-mentioned clinical case experiencing persistent physical symptoms, we could argue that the symptoms are not simply attributes of sensations but are symptoms in and of themselves. Crucially, through the lens of active inference, patients do not simply interpret their bodily sensations, they actively create their own sensoria [9]. Their explanations for what she is experiencing are the best explanations available at the time. They result from meticulously constructed narratives spanning years of participation with her own body and health care professionals. One’s pledge to the narrative that they suffer from persistent pain and other physical symptoms is not based on the content of their prior beliefs but rather on their commitment to the narrative. In contrast, their inability to divert their attention away from the sensory evidence necessitates this type of explanation [3]. The ability to ignore, attend away from, or selectively attenuate different sources of sensory evidence is impaired in chronic pain sufferers. Chronic pain sufferers lack precision in the context of selective attention, and therefore they cannot attenuate or amplify sensations [3,9,56].

## 4. Final Remarks

Appropriate verbal and non-verbal communication, principally touch-based strategies, lay the groundwork for developing trust, compliance, cooperation, and prosocial communication [44,57]. A robust therapeutic alliance centred on cooperative communication and shared narratives and the appropriate and effective use of touch and hands-on care enable patients to contextualize their lived experiences. Hands-on care is crucial because it effectively communicates one’s cognitions and perceptions, provides context and precision, establishes an interpersonal connection, and infers another’s mental states, facilitating biobehavioural synchronization [58,59]. Active inference enables patients and practitioners to disambiguate the mental state of each other [60]. Touch and hands-on care enhance the therapeutic alliance, mental state alignment, and biobehavioural synchrony between patient and practitioner. Therefore, the osteopath–patient dyad provides mental state alignment and opportunities for ecological niche construction. The osteopath aims to modify their patient’s symptoms and promote allostatic regulation during the process of care. We argue that this elicits bidirectional reactions evoked by hands-on experience and reinforced by communicating effectively [61,62]. Developing synchrony between brains [57] and allostatic co-regulation [59,63,64] and thus consolidating interpersonal relationships are arguably possible with osteopathic care characterized by a strong therapeutic alliance [65]. Establishing biobehavioural synchrony is crucial since the intertwined Markov blankets, with reciprocally forecasting sensory experiences produced by the osteopath and patient, lay the foundation for a mutual narrative and epistemic trust, both required for the dissolution of solid beliefs and priors.

Osteopathy is a form of care centred on the person, and as such, osteopaths need to take their modus operandi away from a reliance on etiological models to critically appraising the importance of appropriate forms of communication, the education of patients and affective and cognitive reassurance to deliver psychologically informed osteopathic care [8]. The psychologically informed approach to osteopathic care is bidirectional from the perspective of (En)active inference: (1) when providing novel sensory signals, manual therapy interventions influence the Bayesian inferences from lower to higher levels of the neuronal hierarchy; (2) when updating the generative models, touch associated with appropriate communication, adequate education of the patient, and reassurance modulate top-down predictive processes [44]. As a result of the clinician’s narrative, sensory signals generated by manual therapy interventions will be rapidly interpreted at the lower levels, and possibly self-evidently, regarding the generative models being revised, leading to the ‘flattening of sensory data’ [8]. The active inference cycle can be modulated by psychologically informed osteopathic care, allowing for revised sense-making due to the patient attending to proprioceptive, interoceptive, and exteroceptive signals, simultaneously modifying the active inference cycle. Because of this, it is critical to both attenuate some forms of attention that are orchestrated by the patient’s higher levels of their hierarchical generative models and increase other forms of attention. 

Drawing upon the enactivism and active inference framework, we can improve existing models of osteopathic care by evoking the mechanisms that underpin dyadic exchanges, niche construction, the therapeutic alliance, and the outcomes of person-centred osteopathy. Living organisms must ensure that they only visit their characteristic or expected states to minimize the surprise of their sensory observations, thus maintaining an optimal entropy [32]. Suppose a patient is experiencing chronic pain associated with various psychosocial factors. As an ecological niche, the dyad between the osteopath and the patient gives the two individuals with relevant affordances to promote adaptations and the patient’s ability to regain their agency to undergo their activities of daily living, thus enhancing their health and well-being. Osteopaths should assist their patients in developing a repertoire of attentional deployment that is significantly greater than what is required to reinterpret their interoceptive signals and enable them to regain their capacity to disregard and reassess irrelevant signals to return to a more natural state of being able to render things invisible when necessary. This should enable patients to regain control of their bodies and explore alternative responses to relevant signals emanating from within them. Clinical staff should use comforting, positive communication to help patients make meaningful interpretations of sensory signals and, as a result, revise their generative models. Therefore, psychologically informed osteopathic care can produce experiences similar to mindfulness meditation that reduce the prominence given to high-level prediction errors and, consequently, the top-down attentional focus on bottom-up sensory prediction errors, thus minimizing free energy. 

## Data Availability

Not applicable.
